# Obstetricians’ and Gynecologists’ Communication Practices around Smoking Cessation in Pregnancy, Secondhand Smoke and Sudden Infant Death Syndrome (SIDS): A Survey

**DOI:** 10.3390/ijerph17082908

**Published:** 2020-04-23

**Authors:** Jennah M. Sontag, Binu Singh, Barbara M. Ostfeld, Thomas Hegyi, Michael B. Steinberg, Cristine D. Delnevo

**Affiliations:** 1 SIDS Center of New Jersey, Department of Pediatrics, Robert Wood Johnson Medical School, Rutgers University, New Brunswick, NJ 08901, USA; ostfelba@rwjms.rutgers.edu (B.M.O.); hegyith@rwjms.rutgers.edu (T.H.); 2 Center for Tobacco Studies, Rutgers University, New Brunswick, NJ 08901, USA; steinbmb@rwjms.rutgers.edu (M.B.S.); delnevo@sph.rutgers.edu (C.D.D.); 3 Department of Medicine, Robert Wood Johnson Medical School, Rutgers University, New Brunswick, NJ 08901, USA; 4 Department of Health Behavior, Society & Policy, Rutgers-School of Public Health, Piscataway, NJ 08854, USA

**Keywords:** cessation, secondhand smoke, SIDS, risk communication, obstetricians and gynecologists, pediatrics

## Abstract

Secondhand smoke (SHS) is a potential direct cause of Sudden Infant Death Syndrome (SIDS) among infants. Disparities in SHS exposure and SIDS deaths may be due to inconsistent communication among practitioners about SHS/SIDS risks. In order to assess current SHS/SIDS risks and communication practices and to identify areas of improvement, we conducted a survey of 316 obstetricians and gynecologists (ob/gyns) about the length of time spent having discussions, supplemental materials used, risks covered, cessation, and frequency of discussions. Most (55.3%) reported spending 1–4 min discussing risks/cessation. Nearly a third reported not using any supplemental materials; few used apps (4.4%) or videos (1.9%). Assisting patients with steps toward cessation was infrequent. Few ob/gyns had discussions with patients immediately postpartum. Only 51.9% strongly agreed that they felt sufficiently informed about SHS/SIDS risks to educate their patients. The communication by ob/gyns of SHS/SIDS risk varies greatly and presents opportunities for improvement. Each additional minute spent having discussions and the use of supplemental materials, such as apps, may improve communication effectiveness. The discussion of smoking behaviors immediately postpartum may help to prevent smoker relapse. An increased awareness of statewide cessation resources by ob/gyns is needed to assist patients with cessation. The development of standardized risk messaging may reduce the variation in communication practices among ob/gyns.

## 1. Introduction 

Sudden Unexpected Infant Death (SUID), which comprises Sudden Infant Death Syndrome (SIDS), accidental suffocation and strangulation, and unknown causes, results in nearly 3700 infant deaths every year in the US [1] and is a leading contributor to infant mortality. One of the most significant risk factors, and potentially a direct cause of SIDS, is secondhand smoke (SHS), either from a pregnant smoker or from the environment [2,3]. There is a strong, dose-dependent relationship between smoking during pregnancy and SUID [4], and a number of mechanisms exist whereby maternal smoking increases the risk for SIDS, including the influence of maternal cigarette smoking on reduced infant arousability from sleep [5,6,7,8]. It is estimated that every year 800 infant deaths, or 22% of SUID cases in the US, can be considered attributable to maternal smoking [4]. In addition, birth defects and childhood health problems are associated with exposure to SHS [9].

There are racial disparities in the rates of overall infant mortality in the USA and in the rates specific to SUID [10]. Disparities due to many environmental and social factors have been explored in this context [11], as well as disparities in the degree of compliance with the American Academy of Pediatrics guidelines on reducing the risk of SIDS and other sleep-related infant deaths [12,13]. Among these is a disparity in addressing the dangers of exposure of infants to tobacco smoke [14].

Practitioner communication with patients about the risks of SHS/SIDS may not address all risk factors [15] and varies greatly [16], as does the practitioners’ knowledge of these risks [17,18]. There are also inconsistencies in hospital policy to ensure that new mothers are properly informed about these risks postpartum [19]. Obstetricians provide prenatal care and/or serve as birth attendants to nearly 90% of all births in the US [20,21]. Therefore, we sought to conduct a baseline assessment of the current communication practices among obstetricians and gynecologists (ob/gyns) around smoking cessation options, the risks of SHS, the association between SHS and SIDS, and other risk factors for SIDS. This information can be used to determine areas of improvement for overall best practices and to inform how future SHS/SIDS communication interventions should be developed and implemented.

## 2. Materials and Methods 

This study was part of a repeated, cross-sectional survey exploring physicians’ knowledge, attitudes, and communication about e-cigarettes as well as tobacco cessation with their patients and targeted four specialties: family medicine, internal medicine, pediatrics, and ob/gyns. Each specialty had 750 physicians sampled. The survey sample was compiled from the American Medical Association’s (AMA) Physician Masterfile through the certified vendor Medical Marketing Service (MMS). The Physician Masterfile is the most comprehensive database of physicians and includes data on over 900,000 physicians, residents, and trainees. Physicians in the database include AMA members as well as nonmembers, and the database includes all medical specialties and practice types. The sample of 750 physicians was randomly pulled from the database by MMS. Survey fielding occurred from April−July 2019. A team of research associates mailed physicians an invitation letter that contained an upfront incentive and a link to complete the survey online. Every two weeks thereafter, a reminder mailing was sent that contained the survey link, for a total of four mailings. All surveys were completed online and were anonymous. For ob/gyns only, the core survey was supplemented with a SHS/SIDS module to assess their communication practices about the risks of SHS to infants, the risk factors for SIDS, including smoking and noncompliance with infant safe-sleep practices, and smoking cessation. Our analytic sample for this study is based on the 316 ob/gyns who completed the SHS module. Rutgers Institutional Review Board approved the study procedures.

### 2.1. Measures

Participants were asked how many minutes, on average, they spent talking about the risks of SHS and/or cessation options, and which supplemental materials they provided to patients during these discussions (printed materials (i.e., flyers, handouts), websites, phone numbers, videos, apps, neither, other).

Respondents indicated when they discussed (a) their patients’ smoking habits, (b) the smoking habits of those who live with the patient, and (c) SHS risks, as well as when they (d) had follow-up discussions about these topics and (e) inquired about their patients’ quitting progress (before pregnancy, first trimester, second trimester, third trimester, immediately following delivery, during postpartum visits, I do not inquire about this topic).

Participants were then asked how often they asked their patients questions about their smoking behavior and steps toward cessation (seven items, from never, rarely, some of the time, most of the time, always) (see items in Table 1).

Respondents indicated the extent to which they agreed/disagreed with potential barriers (see to providing cessation treatment (eight items, from strongly disagree, somewhat disagree, somewhat agree, strongly agree. Responses were collapsed into two categories for analysis: at least somewhat disagree vs. at least somewhat agree (see items in Figure 3). 

Participants answered questions about which health risks of SHS they discussed with their patients (preterm birth, low birth weight, birth defects, SIDS) and which risk factors for SIDS, other than smoking, they discussed (placing the infant on back to sleep; avoiding bed-sharing in favor of room sharing; and eliminating pillows, quilts, stuffed animals, bumpers, and other loose and soft bedding from the crib). They were asked whether they discussed strategies to reduce SHS exposure to infants among patients who continued to smoke postpartum.

Then, participants were asked about the extent to which they agreed/disagreed with several statements about their role and ability in the provision of information about smoking risks to their patients (three items, from strongly disagree, somewhat disagree, somewhat agree, strongly agree).

Participants were asked about whether their patients had asked about e-cigarettes in the past 30 days and whether they recommended smokers to switch to e-cigarettes. 

Lastly, participants provided demographic information, including age, gender, and race, and indicated whether they had ever tried an e-cigarette and whether they had smoked at least 100 cigarettes in their lifetime. The standard definition of a “smoker”, as defined by the National Health Interview Survey from the Center for Disease Control and Prevention, is 100+ cigarettes smoked in a lifetime [22]. A physician’s smoking status has been shown to significantly affect the knowledge, attitude, and practices related to smoking cessation counselling [23]. 

### 2.2. Data Analysis

The means and frequencies were calculated for demographics. One-way ANOVA tests were used to examine group differences and Tukey post-hoc tests for pairwise comparisons. Adjusted Odds Ratios (AORs) were calculated to examine smoking cessation communication practices among age subgroups. The analyses were conducted using IBM SPSS 25 (Armonk, NY, USA) with a significance level of 0.05.

## 3. Results

The demographic breakdown of the 316 supplemental survey respondents (a 91% response rate within the 54.1% overall response rate of ob/gyn participants, *n* = 349) was as follows: 56.6% were female, 73.3% were white, 45.7% were 33–49 years old, 43.1% were 50–65 years old, and 11.2% were 66+ (M = 52.0 years, SD = 10.5 years). The majority of participants (87.2%) had not smoked 100 cigarettes or more in their lifetime, and 97.1% had never tried an e-cigarette. No significant differences in characteristics of respondents and non-respondents were found for data that were available for both groups.

The majority of respondents (55.3%) reported spending 1–4 min discussing SHS risks/cessation with their patients, while 37.4% spent 5–8 min, 5.4% spent 9+ min, and 1.9% did not have these discussions.

**Figure 1:**
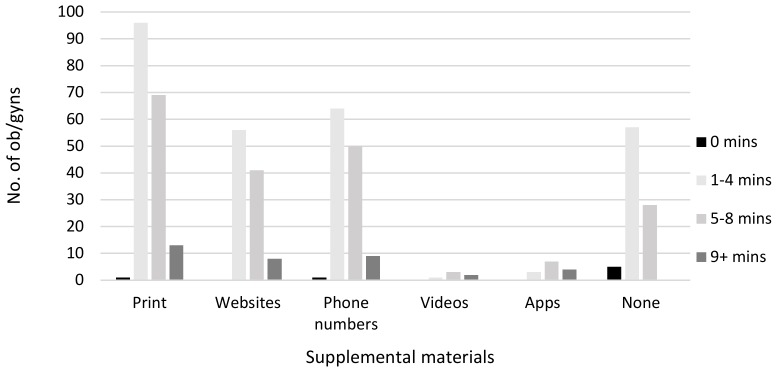
Obstetrician and gynecologist (ob/gyn) use of supplemental materials by minutes spent discussing SHS risks/cessation with patients.

To supplement discussions, 57% of respondents used printed material (e.g., flyers, handouts, etc.), followed by phone numbers (39.2%), websites (33.2%), or nothing at all (28.5%). Few respondents reported using apps (4.4%) or videos (1.9%). Significant differences in video use (*F*(3309) = 3.68, *p* < 0.01) and app use (*F*(3309) = 6.43, *p* < 0.001) were found. Tukey post-hoc tests revealed that significantly more respondents who spent 9+ min having discussions used videos and apps compared to those who spent 1–4 min (videos: *p* < 0.01, apps: *p* < 0.001) or 5–8 min (videos: *p* < 0.05, apps: *p* < 0.01) in discussion. Several respondents indicated providing supplemental materials even though they did not have discussions (see Figure 1).

**Figure 2:**
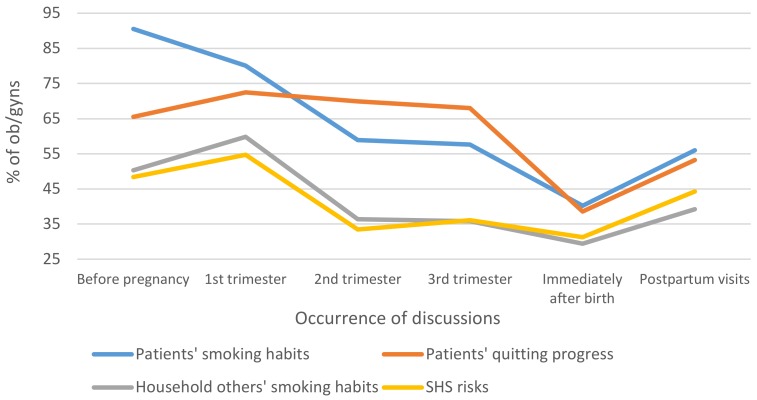
Occurrence of smoking-related discussions.

Initial discussion about patients’ smoking habits occurred before pregnancy for 91% of respondents, while 51.9% inquired about other household smokers, and 51.1% discussed SHS risks before pregnancy. The majority of respondents indicated having follow-up discussions within the first trimester of pregnancy, and a few discussed these topics immediately after childbirth (29.4%, other household smokers; 31.3%, SHS risks) (see Figure 2).

The majority of respondents reported discussing smoking habits and cessation with their patients most or all of the time (Table 1).

However, assisting patients with steps toward cessation was less frequent. Among all respondents, 77.5% discussed medication options (nicotine replacement therapy) for cessation treatment at least some of the time, and the odds of doing so were significantly greater among 50–65 year-olds than among 33–49 year-olds (Adjusted Odds Ratio (AOR) = 2.38, 95% Confidence Interval (CI): 1.24, 4.57, *p* < 0.01) (Table 2). Also, a difference in the frequency with which respondents encouraged patients to set a quit date was found (*F*(3309) = 11.24, *p* < 0.001); respondents who spent 9+ min discussing smoking risks/cessation with their patients more frequently encouraged them to set a quit date compared to those who spent only 1–4 min in discussion (*p* < 0.05).

**Figure 3:**
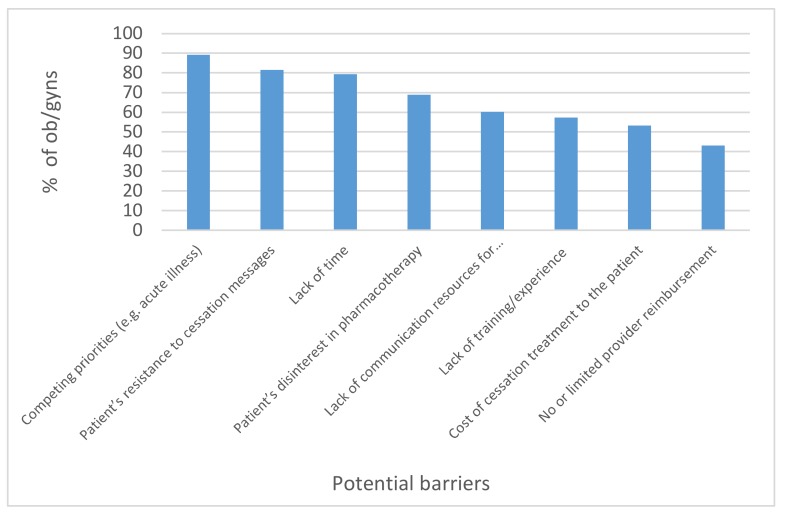
Percentage of ob/gyns that at least somewhat agree with each potential barrier to providing cessation treatment (*n* = 316).

The majority of respondents (89.2%) perceived competing priorities to be a common barrier to providing cessation treatment to patients, followed by the barrier of patients’ resistance to cessation messages (81.6%) and lack of time (79.4%) (see Figure 3).

Among all respondents, 79.4% at least somewhat agreed that lack of time is a barrier to providing cessation treatment. The odds of this belief were significantly greater among 33–49-year-olds (AOR = 6.28, 95% CI: 2.50, 15.80, *p* < 0.001) and 50–65-year-olds (AOR = 3.72, 95% CI: 1.61, 8.59, *p* < 0.01) than those 66+ years old. (Table 2). A significant difference in the perception that a lack of training was a barrier was also found (*F*(3308) = 2.83, *p* < 0.05). Significantly more ob/gyns who spent 1–4 min having discussions, compared to 9+ min, perceived lack of training as a barrier (*p* < 0.05).

During discussions about SHS risks, 84.5% mentioned preterm birth, 89.6% mentioned low birth weight, 78.5% discussed the risk of SIDS, and 76.5% discussed ways to reduce exposure of infants to SHS among patients that smoked postpartum. In addition to SHS, the majority discussed other risk factors of SIDS, including noncompliance with placing infants on their back to sleep (82%), bed-sharing (82.6%), or eliminating all objects from the crib (83.9%). Only 39.6% discussed the risk of birth defects associated with SHS exposure.

While 78.8% strongly agreed that it is their role to discuss the risks of smoking around infants with their patients, only 51.9% strongly agreed that they felt sufficiently informed to educate their patients.

Lastly, in conversations about e-cigarettes, 50.3% reported instances of patients who had asked about e-cigarettes in the past 30 days, and 13.7% had recommended for patients who smoked to switch to e-cigarettes. The odds of recommending smokers to switch to e-cigarettes was significantly greater among 50–65 year-olds than 33–49 year-olds (AOR = 2.82, 95% CI: 1.29, 6.17, *p* < 0.01) (Table 2). 

## 4. Discussion

This survey of the current SHS/SIDS risk communication practices by ob/gyn practitioners reveals several opportunities for improvement. One area of concern is the amount of time that practitioners spend having discussions. Public Health Service Guidelines suggest that brief physician interventions of less than three minutes have shown a statistically significant benefit for cessation [24]. However, this may not be enough time to cover SHS/SIDS risks and to assist parent smokers with steps toward cessation treatment (e.g., establishing quit dates, connecting them with quit resources). Despite recent research that has shown the effectiveness of treatment interventions among parent smokers in a clinical setting, few physicians reported helping patients in this way [25]; furthermore, another study found that only 3.5% of patients received this type of assistance in clinical settings [26]. Although lack of time is perceived to be a potential barrier to discussing cessation treatment, each additional minute spent delivering interventions increases effectiveness because of the dose–response relationship between intervention time and cessation [24].

The use of traditional printed material instead of materials that utilize modern technology, such as videos and apps, is a missed opportunity. The prevalence of mobile phone ownership among new parents indicates that the use of health-related apps is increasing [27,28,29,30,31]. For many new mothers, using a mobile phone is the only means by which they access infant-related health information online [32]. Printed materials that are not reviewed during office visits are likely to be discarded or ignored by patients, especially those with literacy or comprehension challenges, as only an estimated 12% of individuals have proficient health-literacy skills [33]. Videos and apps can be repeatedly accessed after visits and have the potential for widespread dissemination, such as to those without access. 

The finding that most ob/gyns first inquire about their patients’ smoking habits before pregnancy is encouraging, since research demonstrates that smoking before pregnancy can lead to SUID [4]. However, physicians should inquire about patient smoking habits during every visit, because each additional cigarette smoked during pregnancy increases the risk of SIDS [4]. The American Academy of Pediatrics’ guidelines urge against smoking near pregnant women and infants and encourage setting strict rules for smoke-free homes and cars and eliminating SHS in areas where children spend time [12]. Despite this, only 59.8% of ob/gyns discuss the smoking habits of other household smokers with their patients during the first trimester, when most ob/gyns discuss this topic (see Figure 2). When addressing SHS risks, ob/gyns should specifically and more frequently recommend that patients set rules for smoke-free environments, especially postpartum. 

The lowest number of ob/gyns indicated having follow-up discussions immediately following childbirth. Because up to 85% of pregnant smokers who quit during pregnancy relapse after delivery [34], the topics of SHS risks and remaining smoke-free should be reiterated before hospital discharge. 

The low number of ob/gyns who reported assisting their patients with steps toward cessation may be explained by their perceptions of related barriers, highest among them being a lack of time and competing priorities. Even though every state has a telephone quitline and many states have face-to-face treatment options, 60% indicated that a lack of resources was a barrier, which indicates the need for better awareness among ob/gyns of the quit resources available. Although a smaller number of ob/gyns agreed that a lack of experience or training was a barrier, still more than half believed this to be the case. The finding that significantly more ob/gyns who spent only 1–4 min in discussion, versus 9+ min, indicates a lack of training as a barrier and may explain why they only spend a few minutes on these topics. The patient’s resistance to cessation messages was also perceived to be a barrier by most respondents, implying that communication should include strategies for smokers to reduce SHS exposure to children.

While the majority of ob/gyns discuss SHS risks, slightly fewer included the risk of SIDS in these discussions. Most follow the American Academy of Pediatrics’ recommendations to inform parents of safe sleep practices and the benefits of breast feeding (even among women who smoke), as both have been shown to reduce the risk of SIDS [35]. However, fewer ob/gyns discuss the connection between SHS and SIDS. Further, only about 40% present the risk of birth defects associated with SHS. This may be due to a lack of knowledge, which is reflected by only half of the physicians indicating that they felt sufficiently informed about these risks to educate their patients. Because smoking during and/or after pregnancy is often used to cope with the stressors associated with a new infant [36], physicians should provide information about other coping mechanisms and resources, such as social support networks, both through interpersonal relationships [37] and social media [38].

The finding that 50–65-year-old physicians, compared to younger physicians, were more likely to discuss cessation medication options and recommend smokers to switch to e-cigarettes demonstrates the need for standardized messaging. Smokers should receive the same information about cessation-related options, regardless of the age of their physician. Aside from a lack of physician training, a lack of clear hospital policy to educate patients may also contribute to these differences in approaches to cessation.

Although the effects of electronic nicotine delivery systems (ENDS) aerosol exposure on SIDS in humans are unknown, there is animal and epidemiological evidence that nicotine exposure (as found in ENDS aerosol), both pre and postnatally, could impact the risk of SIDS [4,39,40]. Emissions from ENDS are not benign, especially in indoor locations, and could present potential health hazards to bystanders, such as infants. It is prudent to control use in these settings where vulnerable individuals are exposed [41]. In addition, maternal vaping has been shown to be associated with an increased risk of restricted fetal growth [42], and infants that are small for gestational age are at a higher risk for SIDS [43]. In this way, the impact of maternal vaping on the risk of SIDS may be mediated by infants that are small for gestational age due to maternal vaping.

From 9% to 13% of USA births are attended by a midwife [20,21]. A need for increased participation of this provider group in educating families about smoking has been demonstrated [44].

It is acknowledged that there are limitations to this study because, although it was a national cross-sectional survey, the sample size was relatively small and may not be representative of all ob/gyns. Regional representations of the ob/gyn participants of the larger survey were similar to those of the overall ob/gyn sample; however, these data for the ob/gyn supplemental survey participants were not collected, so regional differences in outcome could not be analyzed. In addition, given that a small subset of ob/gyns did not complete the SHS module, there may be some nonresponse bias.

## 5. Conclusions

Provision of communication about SHS/SIDS risks should be optimized to be time-efficient and include ways to reduce SHS exposure to infants and information about the risks of e-cigarettes. Perhaps through the use of modern communication channels, such as videos and apps, this can be accomplished. Communication through these channels could reduce time-constraint barriers and provide standardized information delivery for all patients. Furthermore, updating and standardizing training and education appear to be equally necessary to increase practitioners’ awareness of the risks of SIDS and birth defects associated with SHS exposure.

Future research should continue to examine the communication between ob/gyns and their patients about the risks of SHS/SIDS and further expanded to encompass such topics as racial disparities in receiving education and effective communication strategies and materials.

## Figures and Tables

**Table 1:** Frequency of discussions related to smoking/cessation, *n* (%) (*n* = 316).

Measures	Never	Rarely	Some of the Time	Most of the Time	Always
Ask your patients if they smoke	0 (0)	4 (1.3)	11 (3.5)	62 (19.6)	239 (75.6)
Advise smokers to quit	1 (0.3)	3 (0.9)	16 (5.1)	88 (27.8)	208 (65.8)
Ask smokers if they are interested in quitting	2 (0.6)	12 (3.8)	50 (15.8)	106 (33.5)	146 (46.2)
Encourage smokers to set a quit date	14 (4.4)	57 (18)	105 (33.2)	78 (24.7)	62 (19.6)
Discuss medication options (nicotine replacement therapy)	11 (3.5)	60 (19)	117 (37)	79 (25)	48 (15.2)
Refer interested smokers to cessation treatment	32 (10.1)	65 (20.6)	81 (25.6)	69 (21.8)	68 (21.5)
Follow up with a letter or call	138 (43.7)	118 (37.3)	33 (10.4)	18 (5.7)	8 (2.5)

**Table 2:** Association between age subgroups of ob/gyns, and communication practices and perceptions.

Age Subgroup	Prevalence and Odds of Discussing Medication Options (Nicotine Replacement Therapy) for Cessation Treatment at Least Some of the Time *					Prevalence and Odds of at Least Somewhat Agreeing that Lack of Time Is a Barrier to Providing Cessation Treatment					Prevalence and Odds of Recommending Smokers Switch to E-Cigarettes				
Years old	%	AOR	95% CI		*p*-Value	%	AOR	95% CI		*p*-Value	%	AOR	95% CI		*p*-Value
33–49	71.2	ref				84.9	6.28	2.50	15.80	<0.001	8.7	ref			
50–65	83.8	2.38	1.24	4.57	<0.01	79.4	3.72	1.61	8.59	<0.01	20.6	2.82	1.29	6.17	<0.01
66+	85.3	2.73	0.92	8.08	0.069	52.9	ref				8.8	0.96	0.24	3.83	0.949

Adjusting for gender and race/ethnicity. * Responses to frequency of discussing medication options for cessation treatment (never, rarely, some of the time, most of the time, always) were collapsed into two categories for analysis: never/rarely vs. at least some of the time. AOR = Adjusted Odds Ratio. CI = Confidence Interval. ref = reference group.
